# Benchmarking Audio Signal Representation Techniques for Classification with Convolutional Neural Networks

**DOI:** 10.3390/s21103434

**Published:** 2021-05-14

**Authors:** Roneel V. Sharan, Hao Xiong, Shlomo Berkovsky

**Affiliations:** Australian Institute of Health Innovation, Macquarie University, Sydney, NSW 2109, Australia; hao.xiong@mq.edu.au (H.X.); shlomo.berkovsky@mq.edu.au (S.B.)

**Keywords:** convolutional neural networks, fusion, interpolation, machine learning, spectrogram, time-frequency image

## Abstract

Audio signal classification finds various applications in detecting and monitoring health conditions in healthcare. Convolutional neural networks (CNN) have produced state-of-the-art results in image classification and are being increasingly used in other tasks, including signal classification. However, audio signal classification using CNN presents various challenges. In image classification tasks, raw images of equal dimensions can be used as a direct input to CNN. Raw time-domain signals, on the other hand, can be of varying dimensions. In addition, the temporal signal often has to be transformed to frequency-domain to reveal unique spectral characteristics, therefore requiring signal transformation. In this work, we overview and benchmark various audio signal representation techniques for classification using CNN, including approaches that deal with signals of different lengths and combine multiple representations to improve the classification accuracy. Hence, this work surfaces important empirical evidence that may guide future works deploying CNN for audio signal classification purposes.

## 1. Introduction

Sensing technologies find applications in detecting and monitoring health conditions. For example, audio signals, such as speech, can be useful in detecting anxiety [1] and commanding wheelchair movement [2], acoustic event recognition in elderly care [3], and respiratory sounds in detecting respiratory diseases [4].

Convolutional neural network (CNN) is an established image classification technique that has outperformed conventional methods in various applications, such as in handwritten digit recognition [5] and on the ImageNet dataset [6] containing various image categories. Although deep learning methods, and CNN in particular, were originally designed for large datasets, techniques such as data augmentation [7], transfer learning [8], and regularization [9] have allowed their extension to small datasets with encouraging results [10,11,12,13].

Due to such advancements, the robustness of CNN, and forgoing the need for complex feature engineering and extraction required by conventional methods [14,15,16], it was not long before CNN was adopted in audio signal classification tasks, achieving results superior to conventional techniques [17,18,19]. However, unlike in image classification, where raw images can be used as a direct input to the CNN, audio signal classification using CNN presents several practical challenges.

Firstly, the raw time-domain signals can be of a varying length [20,21]. Secondly, using time-domain signals for classification with CNN has generally failed to surpass the accuracy achieved with frequency-domain analysis [22,23,24,25], which required signal transformation. Finally, feature combination, a commonly used technique for improving the classification performance using conventional classification methods, is not as straightforward to apply to CNN.

Audio signal classification finds various applications and there has been a growing interest in audio signal classification using deep learning and CNN. The advancements in CNN techniques have been covered in several papers [26,27,28,29,30]. Furthermore, while numerous signal representation techniques have been proposed for audio signal classification using CNN, there is a lack of literature critically reviewing and evaluating the various signal representation techniques to be used in conjunction with CNN.

The main contribution of this work is to *overview and benchmark several popular audio signal representation techniques for classification using CNN*. In particular, we focus on time-frequency image representations, time-frequency image resizing techniques to deal with signals of varying lengths, and strategies to combine the learning from different signal representations. The benchmarking results bring to the fore interesting findings about the contribution of these signal representations to the CNN classification accuracy. Our work provides valuable insight for machine learning researchers deploying CNN for audio signal classification tasks.

## 2. Literature Review

CNN was originally conceived as an image classification technique and one of the challenges in classifying audio signals using CNN has been to find an appropriate image-like representation of the signal. Time-frequency representation of the audio signals is a common approach to forming this image-like representation. Another requirement of CNN is that input images are expected to have the same dimension. This presents another challenge in applications, where the signals are of a different duration, such as in isolated acoustic event and word detection.

Various time-frequency image formation techniques have been proposed for CNN. The conventional spectrogram representation, formed using short-time Fourier transform (STFT) [31,32], is still widely used, such as in speech emotion recognition [33] and spoken digit recognition [25].

This approach, however, has disadvantages. While having a large number of points in computing the Fourier transform can adequately reveal the unique frequency characteristics, it increases the computational costs of CNN. A smaller number of points, on the other hand, may not accurately capture the unique frequency characteristics, resulting in poor classification performance.

A widely used alternative is to use a large transform length and then frequency filterbanks to compute the filterbank energies in various frequency subbands. Two such filters are the moving average filter [34] and mel-filter. The spectrogram representation formed using the moving average filter is called the moving average spectrogram or smoothed-spectrogram [35,36].

The mel-filter, as used in computing mel-frequency cepstral coefficients (MFCCs) [37], has frequency bands equally spaced on the mel-scale [38] resembling the way humans perceive sound. Representations using the mel-scale are popular for use with CNN, as seen in the 2016 Challenge on Detection and Classification of Acoustic Scenes and Events (DCASE) [39]. The image-like representations used with mel-scale are MFCCs, which sometimes include the derivatives (delta and delta-delta coefficients [40]) and the log energies or mel-spectrogram.

In addition, gammatone filters, which model the human auditory system, are used for forming the time-frequency representation of audio signals [41,42], called gammatone-spectrogram or cochleagram. Constant-*Q* transform (CQT) [43] is another technique for frequency transformation of signal and this is used in time-frequency representation of audio signals [44,45].

Furthermore, in isolated word or acoustic event classification, the duration of the signals can vary greatly. For signals of unequal length, dividing the signal into an equal number of frames is a common approach to obtain the same number of units in the time-frequency representation [18,36]. However, dividing the signal into an equal number of frames can result in small frames for short signals, possibly making it difficult to capture unique frequency characteristics. An alternative can be to divide the signal into frames of a fixed length, but this will result in a different number of frames for different signals. This was a commonly used technique in computing conventional features, such as MFCCs, whereby the final feature vector could be represented using statistical features, such as mean and standard deviation, computed across all frames. To get an equal sized time-frequency representation, techniques such as image resizing [41,44,46], zero-padding [47,48], and cropping [49] have been deployed.

Moreover, feature combination has been a common practice in various audio classification applications. This allows fusing information acquired from different signal processing techniques and potentially achieving an improved classification performance. Most techniques revolved around combining MFCC features with features such as wavelets, temporal and frequency descriptors, time-frequency image descriptors, and matching pursuit [50], using classifiers such as support vector machine (SVM) and Gaussian mixture model (GMM) [51,52,53,54].

With CNN specifically, the concept of feature combination can be realized by using multiple signal representations for classification. Different filters capture frequency characteristics at different centre frequencies and bandwidths. As such, it might be possible to improve CNN using various signal representations. A number of strategies have been proposed to combine the learning from multiple representations [24,45,55,56]. Broadly, the methods can be categorized as early-fusion, mid-fusion, and late-fusion [57,58,59,60]. These refer to the classification stage at which the information is combined, such as combining the inputs to the CNN in early-fusion, combining the weights of the middle layers of the CNN in mid-fusion and combining the CNN outputs in late-fusion.

## 3. Audio Signal Representation Techniques

**Figure 1:**
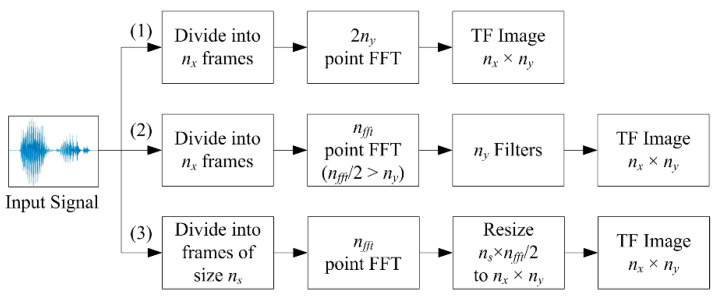
Overview of same sized time-frequency (TF) image formation techniques.

We discuss the implementation techniques for various time-frequency representations for use with CNN, approaches to deal with signals of different lengths, and signal representation fusion techniques. An overview of the common techniques for forming the time-frequency representations is given in Figure 1. The target time-frequency image dimension is $n_{x}$ × $n_{y}$ where $n_{x}$ denotes the number of time windows along the x−axis and $n_{y}$ the number of frequency components along the y−axis. The procedure for computing these time-frequency representations is detailed in the following subsections.

### 3.1. Time-Frequency Image Representations

In forming the conventional spectrogram (Path (1) in Figure 1), all the signals are divided into $n_{x}$ frames and a $2n_{y}$-point discrete Fourier transform (DFT) is computed. Taking the single-sided spectrum results in a time-frequency representation of size $n_{x}$ × $n_{y}$. The computation of STFT is given as

(1) $$X\left(k,r\right)=\sum_{n=0}^{N-1}x\left(n\right)w\left(n\right)e^{\frac{-2\pi ikn}{N}},\;\begin{array}{cc} & k=0,\ldots ,N-1 \end{array}$$

where N is the length of the window function, x(n) is the time-domain signal, X(k,r) is the kth harmonic for the rth frame, $F_{s}$ is the sampling frequency, and w(n) is the window function.

The spectrogram values are obtained from log of the DFT values’ magnitude as

(2) $$S\left(k,r\right)=\log{\left|X\left(k,r\right)\right|}^{2}.$$

In forming the smoothed-spectrogram or mel-spectrogram (Path (2) in Figure 1), the filterbank output of the fth filter is given as

(3) $$E\left(f,r\right)=\sum_{k=0}^{\frac{N}{2}-1}V\left(f,k\right)\left|X\left(k,r\right)\right|,\begin{array}{cc} & f=1,2,\ldots ,F \end{array}$$

where V(f,k) is the normalized filter response of the moving average filter or mel-filter and F is the total number of filters. The log representation can be computed using (2).

The impulse response of the gammatone filter used for forming the cochleagram representation is given as

(4) $$h\left(t\right)=At^{j-1}e^{-2\pi Bt}\cos\left(2\pi f_{c}t+\phi \right)$$

where A is the amplitude, j is the order of the filter, B is the bandwidth of the filter, $f_{c}$ is the centre frequency of the filter, ϕ is the phase, and t is the time [61].

The gammatone filters are equally spaced on the equivalent rectangular bandwidth (ERB) scale [61].The three commonly used ERB filter models are given by Glasberg and Moore [62], Lyon’s cochlea model [63], and Greenwood [64]. Implementation of a fourth order gammatone filter with four filter stages and each stage being a second order digital filter is described in [65] and a MATLAB implementation is provided in [66].

### 3.2. Time-Frequency Image Resizing Techniques

Image scaling or resizing using interpolation is a commonly used technique in digital image processing which can be applied to time-frequency image as well (Path (3) in Figure 1). This can be achieved by convolving an image with a small kernel, such as nearest-neighbor, bilinear, bicubic, Lanczos-2, and Lanczos-3 [41,46,67,68].

Nearest neighbour interpolation selects the value of the nearest neighbouring point,

(5) $$R_{NN}\left(x,y\right)=S_{\left[x\right]\left[y\right]}$$

the kernel for which in one dimension is given in [69] as

(6) $$k\left(x\right)=\{\begin{array}{ll} 1, & \left|x\right|<0.5\\ 0, & otherwise. \end{array}$$

Bilinear interpolation, an extension of a linear interpolation in the x and y directions, is given as

(7) $$R_{BL}\left(x,y\right)=a_{0}+a_{1}x+a_{2}y+a_{3}xy$$

where the coefficients are determined from the four nearest neighbours of (x,y) and implemented using a triangular kernel as

(8) $$k\left(x\right)=\{\begin{array}{ll} 1-\left|x\right|, & \left|x\right|<1\\ 0, & otherwise. \end{array}$$

Bicubic interpolation resamples 16 neighbouring pixels as

(9) $$R_{BC}\left(x,y\right)=\sum_{i=0}^{3}\sum_{j=0}^{3}a_{ij}x^{i}y^{j}$$

where the coefficients are determined from the sixteen nearest neighbours of (x,y) and apply convolution with the kernel proposed in [70]

(10) $$k\left(x\right)=\{\begin{array}{ll} \frac{3}{2}{\left|x\right|}^{3}-\frac{5}{2}{\left|x\right|}^{2}+1, & \left|x\right|\leq 1\\ -\frac{1}{2}{\left|x\right|}^{3}+\frac{5}{2}{\left|x\right|}^{2}-4\left|x\right|+2, & 1<\left|x\right|\leq 2\\ 0, & otherwise. \end{array}$$

The Lanczos kernel is a normalized sinc function [71] windowed by the sinc window, which can be equivalently written as

(11) $$L\left(x\right)=\{\begin{array}{ll} 1, & x=0\\ \frac{a\sin\left(\pi x\right)\sin\left(\pi x/a\right)}{\pi^{2}x^{2}}, & -a\leq x<a\;\mathrm{and}\;x\neq 0\\ 0, & otherwise \end{array}$$

where a is a positive integer; the kernel is referred as Lanczos-2 when a=2 and Lanczos-3 when a=3 [72].

The Lanczos interpolation is computed as

(12) $$R_{L}\left(x\right)=\sum_{i=\left\lfloor x\right\rfloor -a+1}^{\left\lfloor x\right\rfloor +a}S_{i}L\left(x-i\right)$$

where ⌊x⌋ is the floor function of x, a is the filter size, and $S_{i}$ is a one dimensional signal [69]. The Lanczos kernel in two dimensions is given as L(x,y)=L(x)L(y).

### 3.3. Combination of Signal Representations

**Figure 2:**
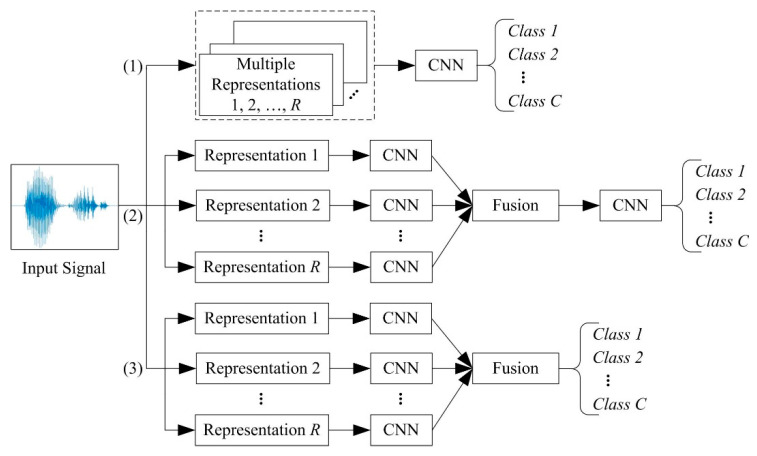
Overview of fusion techniques for learning from multiple representations of the same signal: (1) early-fusion, (2) mid-fusion, and (3) late-fusion.

Three common techniques for fusion of time-frequency image representations—early-fusion, mid-fusion, and late-fusion—are illustrated in Figure 2. According to the early-fusion method (Path (1) in Figure 2), multiple representations of the signal are treated as individual channels, similar to a coloured image, on which a single CNN is trained. This technique could also be referred as a multichannel CNN. According to the mid-fusion method (Path (2) in Figure 2), a CNN is trained on each representation of the signal. The activations of all the CNNs are combined and trained in the final layers of the CNN, or in another classifier, to make the final prediction. In late-fusion (Path (3) in Figure 2), CNN outputs trained on the individual representations are fused, e.g., averaging the output score values. The latter two methods could be called multi-input CNN.

## 4. Benchmarking

We evaluate the classification performance of different time-frequency image representation techniques, time-frequency image resizing techniques, and signal representation combination techniques on two audio classification tasks: sound event recognition and isolated word recognition.

### 4.1. Experimental Setup

#### 4.1.1. Datasets

For the sound event recognition task, we use the Real World Computing Partnership Sound Scene Database (SSD) [73]. The subset of the dataset used in this work has 4000 sound event files, 80 files for each of the 50 classes. The signals are sampled at 44.1 kHz with a 16-bit resolution. The average duration of the segmented signals is 0.5905 s. Furthermore, 50 files from each class are used for training and validating the CNN model in five-fold stratified cross-validation and the remaining 30 are used for testing.

For the isolated word recognition task, we use the Speech Commands dataset [74] which is sampled at 16 kHz. All 105,829 utterances across 35 classes, which have duration of 1 s or less, were used together with 4063 samples of an additional *noise* class generated from six long background noise segments. The dataset was divided into training, validation, and test sets as per the dataset annotation. The noise class was randomly split into the training, validation, and test datasets using the 80%-10%-10% ratio. The final dataset is comprised of 88,093 training segments, 10,387 validation segments, and 11,411 test segments.

In the experiments we report the classification accuracy obtained for the validation and test sets. This communicates the ratio between the number of correctly classified sound events or speech commands and the overall number of classifications.

#### 4.1.2. CNN

The CNN architecture and hyperparameter settings for the two datasets are given in Table 1 and Table 2, respectively. A target time-frequency image representation of 32 × 15 (*height* × *width*) is used for the sound event dataset. The CNN architecture deployed is similar to that of [36,41] except for the optimization that is performed using adaptive moment estimation (Adam) [75], which was shown to outperform stochastic gradient descent with momentum [76]. The network has two convolutional layers, each of which is followed by batch normalization [77], rectified linear unit (ReLU) [78], and max pooling [79]. This is followed by a fully connected layer and a softmax layer [76].

For the speech command dataset, we use a target representation of 64 × 64. The CNN architecture is similar to that of [24]. The network has five convolutional layers, each of which is followed by batch normalization and ReLU layers. All ReLU layers, except for the fourth, are followed by a max pooling layer and then the final layers (fully connected and softmax layer).

The early-fusion method is a multichannel CNN, similar to classification of coloured images where the channels represent the R, G, and B image components. For the mid-fusion approach, we found the use of concatenation and addition layers before the fully connected layer to give the best results on the sound event and speech command classification tasks, respectively. The late-fusion approach only requires averaging the probability output of the CNNs trained on the individual representations.

The networks were implemented in MATLAB R2020b and fully trained on AWS using a single NVIDIA V100 Tensor Core GPU.

### 4.2. Classification Results

#### 4.2.1. Time-Frequency Representations

**Figure 3:**
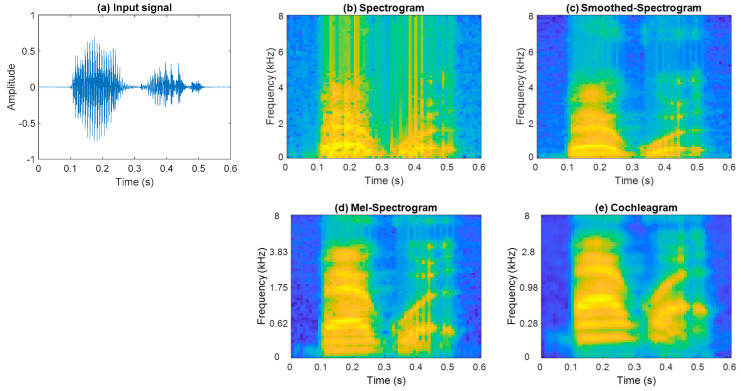
(**a**) Plot of the speech command signal *backward* and its time-frequency representations: (**b**) spectrogram, (**c**) smoothed-spectrogram, (**d**) mel-spectrogram, and (**e**) cochleagram.

For the sound event dataset, to form the spectrogram, each signal is divided into 15 frames with a 50% overlap and DFT is computed using 64 points. Computing the single-sided power spectrum results in a 32 × 15 spectrogram representation. Smoothed-spectrogram and mel-spectrogram use a 1024 point DFT, followed by 32 moving average filters and mel-filters over the single-sided spectrum, respectively, while the cochleagram representation utilises 32 gammatone filters. The speech commands dataset uses a similar approach to form the time-frequency representations. A plot of an example speech command signal *backward* and its four time-frequency representations are shown in Figure 3.

The classification results in Table 3 show that the use of filterbank energies improves the classification accuracy over the conventional spectrogram. For both of the datasets, the highest classification accuracy is achieved using the cochleagram representation. The relative improvement over the test results using spectrogram representation on the sound event and speech command datasets are 5.16% and 2.43%, respectively. The results suggest that, out of the four time-frequency representations considered for the two audio signal classification tasks, the cochleagram offers the best time-frequency representation for classification using CNN. The finer frequency resolution in the lower frequency range offered by the gammatone filter could explain its robustness in modelling the frequency characteristics of speech and sound event signals [54].

#### 4.2.2. Resized Representations

In this case, the spectrogram representation was formed by dividing each signal into frames of 1024 points for sound events and 256 points for the speech commands. As such, the number of frames was different for signals of different lengths. A 1024 point DFT was then computed and the resulting time-frequency representation was resized to 32 × 15 for the sound event dataset and to 64 × 64 for the speech command dataset using interpolation.

The results in Table 4 show that image resizing techniques improve the classification accuracy over the conventional spectrogram representation. The relative improvements in test classification accuracy, with the highest accuracy achieved by the resized spectrogram, are 3.58% and 2.25% on the sound event and speech command datasets, respectively. The best accuracy values are with the bicubic and Lanczos kernel interpolated spectrograms, which could be attributed to their low error in image scaling [41].

#### 4.2.3. Fusion Techniques

Classification results using the combined signal representations are given in Table 5. We consider the combination of smoothed-spectrogram, mel-spectrogram, and cochleagram representations. In both datasets, the test results using the signal representation combination techniques are better than the best performing individual cochleagram representation results shown in Table 3. In addition, the classification accuracy using late-fusion is better than mid-fusion and early-fusion. This suggests that while the performance of CNN can be improved using fusion techniques, best results on the two tasks considered in this work is when CNN is trained independently on each representation and fusion is performed in the end.

## 5. Discussion and Conclusions

This paper reviews and evaluates various audio signal representation techniques for classification using CNN. On the sound events and speech commands classification tasks, we reviewed and evaluated the spectrogram, smoothed-spectrogram, mel-spectrogram, and the cochleagram time-frequency representations. While smoothed-spectrogram and mel-spectrogram improved the classification performance over the conventional spectrogram, *the cochleagram representation produced the best classification* performance.

The conventional spectrogram representation offers linearly spaced centre frequencies. On the other hand, the cochleagram representation utilises a gammatone filter, which mimics the human auditory model. The centre frequencies are nonlinearly spaced, having closely spaced centre frequencies in the low frequency range with narrow bandwidth and widely spaced centre frequencies in the upper frequency range. Speech and sound event signals have more frequency content in the lower frequency range, as seen in Figure 3, which is better modelled by the gammatone filter and, thereby, outperforms the conventional methods.

We also considered image resizing techniques, in order to resize time-frequency representations of signals of unequal length. The classification results using the *bicubic and Lanczos kernel interpolations performed best* and were comparable to what could be achieved using smoothed-spectrogram and mel-spectrogram. These interpolation methods offer a lower discrepancy between the interpolated and exact image [41,80], which could explain their better classification performance.

In addition, three techniques for combining multiple signal representations for classification using CNN were reviewed: early-fusion, mid-fusion, and late-fusion. Signal representation combination using the *late-fusion method produced the best classification* on both the sound events and speech commands datasets.

We note that the validation performance on the sound event dataset is, generally, slightly lower than the test performance which could be attributed to the relatively small dataset. During validation, the network was trained on only 40 samples and validated on 10 samples. Once we had settled on the network architecture and tuned the hyperparameters in validation, we trained the network with all 50 training samples (increase of 25% in data over 40 samples) and evaluated the performance on the test data. The increase in training data by 25% could explain the slightly higher performance on the test dataset.

In this work, we limited the evaluation of the audio signal representation combinations to time-frequency representations. However, the studied techniques can be extended to other representations. For example, feature combination of MFCCs and wavelets [52] and MFCCs and time-frequency features [53,54] produced robust classification performance in audio classification tasks. The fusion-based techniques can be extended to these representations as well.

It should be mentioned that the audio signal representation techniques evaluated in this work are for back-end classification using CNN. There is a growing interest in end-to-end CNN models with raw audio signals as input [81,82,83]. However, a number of these techniques use frequency filters, such as gammatone filters. These are beyond the scope of this work and we plan to study them in the future.

We believe that our findings will be valuable for future works aiming to combine signal processing methods with CNN-based classification tasks. The fact that multiple signal representation methods, datasets, and types of signal were exploited and benchmarked strengthens the validity of our findings and their generalisation potential. Our work surfaces valuable experimental evidence and provides practical guidelines to machine learning researchers, deploying CNN and deep neural networks more generally, to signal classification problems.

## Figures and Tables

**Table 1:** CNN architecture used for the sound event and speech command datasets.

	Sound Event	Speech Command
Image input layer	32 × 15	64 × 64
Middle layers	Conv. 1: 16@3 × 3, Stride 1 × 1, Pad 1 × 1 Batch Normalization, ReLU Max Pool: 2 × 2, Stride 1 × 1, Pad 1 × 1 Conv. 2: 16@3 × 3, Stride 1 × 1, Pad 1 × 1 Batch Normalization, ReLU Max Pool: 2 × 2, Stride 1 × 1, Pad 1 × 1	Conv. 1: 48@3 × 3, Stride 1 × 1, Pad ‘same’ Batch Normalization, ReLU Max Pool: 3 × 3, Stride 2 × 2, Pad ‘same’ Conv. 2: 96@3 × 3, Stride 1 × 1, Pad ‘same’ Batch Normalization, ReLU Max Pool: 3 × 3, Stride 2 × 2, Pad ‘same’ Conv. 3: 192@3 × 3, Stride 1 × 1, Pad ‘same’ Batch Normalization, ReLU Max Pool: 3 × 3, Stride 2 × 2, Pad ‘same’ Conv. 4: 192@3 × 3, Stride 1 × 1, Pad ‘same’ Batch Normalization, ReLU Conv. 5: 192@3 × 3, Stride 1 × 1, Pad ‘same’ Batch Normalization, ReLU Max Pool: 3 × 3, Stride 2 × 2, Pad ‘same’ Dropout: 0.2
Final layers	Fully connected layer: 50 Softmax layer Classification layer	Fully connected layer: 36 Softmax layer Classification layer

**Table 2:** Optimization algorithm and hyperparameter settings for training the CNN.

	Sound Event	Speech Command
Optimization algorithm	Adam	Adam
Initial learn rate	0.001	0.0003
Mini batch size	50	128
Max epochs	30	25
Learn rate drop factor	0.5	0.1
Learn rate drop period	6	20
L2 regularization	0.05	0.05

**Table 3:** Average classification accuracy (in %) of time-frequency image representations.

Signal Representation	Sound Event		Speech Command	
	Validation	Test	Validation	Test
Spectrogram	92.70	93.77	92.33	91.90
Smoothed-Spectrogram	96.48	97.32	93.79	93.41
Mel-Spectrogram	96.45	96.31	93.64	93.64
Cochleagram	**98.35**	**98.61**	**94.33**	**94.13**

**Table 4:** Average classification accuracy (in %) of resized time-frequency image representations.

Signal Representation	Sound Event		Speech Command	
	Validation	Test	Validation	Test
Resized spectrogram (nearest-neighbour)	93.51	94.19	93.20	93.10
Resized spectrogram (bilinear)	95.71	96.31	**94.10**	93.81
Resized spectrogram (bicubic)	96.02	96.59	94.03	**93.97**
Resized spectrogram (Lanczos-2)	95.75	96.42	93.75	93.77
Resized spectrogram (Lanczos-3)	**97.01**	**97.13**	94.02	93.75

**Table 5:** Average classification accuracy (in %) of signal representation fusion techniques.

Signal Representation Fusion Technique	Sound Event		Speech Command	
	Validation	Test	Validation	Test
Early-fusion	98.42	98.63	94.47	94.29
Mid-fusion	98.48	98.82	94.65	94.49
Late-fusion	**98.64**	**98.83**	**94.86**	**94.80**

## Data Availability

Not applicable.
